# Effect of Monosaccharides Including Rare Sugars on the Bilayer Phase Behavior of Dimyristoylphosphatidylcholine

**DOI:** 10.3390/membranes14120258

**Published:** 2024-12-03

**Authors:** Nobutake Tamai, Mei Kamiya, Nono Kiriyama, Masaki Goto, Kazuhiro Fukada, Hitoshi Matsuki

**Affiliations:** 1 Department of Bioengineering, Division of Bioscience and Bioindustry, Graduate School of Technology, Industrial and Social Sciences, Tokushima University, 2-1 Minamijosanjima-cho, Tokushima 770-8513, Japan; tamai@tokushima-u.ac.jp (N.T.); goto@tokushima-u.ac.jp (M.G.); 2 Department of Applied Life Science, Division of Bioresource Science, Graduate School of Sciences and Technology for Innovation, Tokushima University, 2-1 Minamijosanjima-cho, Tokushima 770-8513, Japan; 3 Department of Applied Biological Science, Faculty of Agriculture, Kagawa University, 2393 Ikenobe, Miki-cho, Kita 761-0795, Japan; fukada.kazuhiro@kagawa-u.ac.jp

**Keywords:** phospholipid, bilayer membrane, phase transition, rare sugar, differential scanning calorimetry

## Abstract

We observed bilayer phase transitions of dimyristoylphosphatidylcholine (DMPC) in aqueous solutions of four kinds of monosaccharides, namely, D-glucose, D-fructose, D-allose and D-psicose, using differential scanning calorimetry (DSC). D-allose (C3-epimer of D-glucose) and D-psicose (C3-epimer of D-fructose) are rare sugars. We performed DSC measurements using two types of sugar-containing sample dispersions of the DMPC vesicles: one is a normal sample dispersion with no concentration asymmetry between the inside and outside of the vesicles and the other is an unusual sample dispersion with a concentration asymmetry. DSC measurements using normal sample dispersions with different sugar concentrations revealed that the temperatures and transition enthalpies of the pre- and main transition of the DMPC bilayer membrane did not significantly depend on the sugar concentration for all monosaccharides. DSC measurements using the unusual sample dispersions demonstrated that the concentration asymmetry caused the splitting of the endothermic peak of the main transition similarly irrespective of the sort of monosaccharides present. From all these DSC results, we conclude that (i) most monosaccharide molecules exist in the bulk water phase, (ii) no specific interaction depending on the molecular structure of each monosaccharide directly occurs between the DMPC and each monosaccharide molecule, and (iii) almost all the effects of the monosaccharides observed in this study are understandable as the general colligative properties of solutions.

## 1. Introduction

Biological membranes are composed of various kinds of biomolecules, such as membrane proteins, phospholipids and sterols, but their common basic structure is provided by a phospholipid bilayer membrane. Phospholipids are typical amphiphilic molecules, and most membrane phospholipids self-assemble to form molecular aggregates with a bilayer structure in the presence of an adequate amount of water. Therefore, the aggregate structure and physicochemical properties of various kinds of phospholipids have been comprehensively studied using a wide range of experimental techniques, such as differential scanning calorimetry (DSC) [1,2], small-angle X-ray scattering [3,4], fluorescence spectroscopy [5,6,7], and NMR [8,9,10], in order to establish a physical and chemical basis for understanding the various biological functions of cell membranes. Dipalmitoylphosphatidylcholine (DPPC) is one of the phospholipids which have been most frequently used in previous membrane studies [11]. It is well known that the DPPC bilayer membrane undergoes several types of changes in its aggregate structure with increasing temperature [1]: a subtransition at ca. 21 °C, a pretransition at ca. 34 °C and a main transition at ca. 42 °C, which correspond to the conversion from a lamellar crystal (L_c_) phase to a lamellar gel (L_β_′) phase, that from the L_β_′ phase to a ripple (P_β_′) phase and that from the P_β_′ phase to a liquid crystalline (L_α_) phase, respectively. In addition, it has also been reported for the DPPC bilayer that a non-bilayer structure called the interdigitated gel (L_β_I) phase is formed at high pressures above ca. 100 MPa [12,13], in which the hydrocarbon chains of the phospholipid molecules within a monolayer constituting a bilayer penetrate alternatively into the hydrocarbon chain region of the other monolayer of the bilayer. Dimyristoylphosphatidylcholine (DMPC) has also often been used in model membrane studies. Since DMPC has two shorter acyl chains than DPPC, all the transition temperatures of the DMPC bilayer membrane are lower compared to those of the DPPC bilayer membrane, though the bilayer phase behavior of both lipids is almost the same [1,2]. In addition, it is also known that certain kinds of phospholipids (e.g., unsaturated phosphatidylethanolamines) can form molecular aggregates with a nonlamellar structure like an inverted hexagonal phase [14,15]. Now that enough knowledge about membrane properties and structure for various kinds of phospholipids is available, we can systematically explain how the variation in the chemical structure of the constituent phospholipid molecule affects the whole membrane properties and structure.

Such artificially created phospholipid bilayer membranes have long been utilized also as model membranes for studying the interaction between a phospholipid bilayer membrane and a small molecule coexisting as a third species. Since phospholipid bilayer membranes generally exhibit several types of phase transitions as described above, the molecular interaction between a phospholipid and a small molecule of the third species can be evaluated as effects on the bilayer phase behavior, or more quantitatively, as variations in the thermodynamic quantities of the bilayer phase transitions, such as a transition temperature and an enthalpy change. This allows us to classify the types of interaction according to the similarity in the effect on the bilayer phase transitions. For example, Jain and Wu reported that the interaction of small molecules with phospholipid membranes can be classified into at least four different types on the basis of the experimental results obtained from DSC measurements [16]. We often use a broad classification consisting of three categories, depending on the interaction of the added substance with the phospholipid bilayer membrane. The first category of our classification includes the small compounds, such as medium- or long-chain fatty acids [17,18], that interact with phospholipid membranes to enhance the gel-phase stability. In the presence of these small molecules, the temperature and enthalpy of the main transition can increase. The small compounds that interact with phospholipid membranes to produce the opposite effect (i.e., decrease in the main-transition temperature and enthalpy) are classified into the second category. Alcohols and anesthetics are typical examples of this category, which cause a decrease in the main-transition temperature and enthalpy [17,19]. The last category contains other small compounds that cannot be classified into either the first or the second category. As is evident from the simplicity of this classification, most of the small molecules included in this category have a propensity to exhibit more complicated effects on the phase transitions of bilayer membranes as compared to the small molecules belonging to the first or the second category. Cholesterol is a typical compound that exhibits such complicated effects. It is well known that cholesterol incorporated into a phospholipid bilayer membrane can induce another intermediate state between the gel and liquid crystalline phases, and that the main transition itself is eventually abolished in the presence of large concentration of cholesterol [20,21,22,23].

Monosaccharides are the simplest carbohydrates and indispensable for maintaining our life as an energy source. They are also known as significant biomolecules that are involved in important biological functions in the form of sugar chains. Although monosaccharides are typical significant biomolecules, relatively few studies have so far been conducted concerning the effect of sugars, including disaccharides, on phospholipid bilayer membranes in the fully hydrated state. In fact, monosaccharides are not included in the comprehensive study by Jain and Wu [16], who examined the effect of more than 90 compounds on the bilayer phase behavior of DPPC. This might be because sugars are recognized in general as protectants that possess an ability to stabilize the membrane structure to protect it from various environmental stresses, such as freezing and desiccation [24,25,26,27], rather than as membrane-active compounds that can significantly alter the structure and properties of phospholipid bilayer membranes in relatively small quantity. Therefore, many previous studies have aimed to elucidate the molecular mechanism of stabilization by sugars. Currently, two hypotheses have been proposed concerning the mechanism of this stabilization: one is commonly known as the water replacement hypothesis (WRH), and the other is often referred to as the hydration force explanation (HFE). In the former hypothesis, the membrane stabilization is explained by the formation of hydrogen bonds between the polar headgroup of the phospholipid molecule and the sugar molecules intercalated in the interfacial region of the bilayer membrane [28,29,30,31]. In the latter, on the other hand, the membrane stabilization is thought to be caused indirectly by an osmotic imbalance and volume effect as a result of the exclusion of sugar molecules from the interfacial region of the bilayer membrane [32,33,34,35]. These two mechanisms, which are still controversial, are fundamentally different in the respect that sugar molecules tend to be partitioned into or excluded from the bilayer membrane [36]. From a phenomenological viewpoint of the effect of sugars on the bilayer membranes, however, we may say that there is a common understanding that the pre- and main-transition temperature slightly increase in the case of multilamellar vesicles [37,38,39,40,41,42] and that the transition enthalpies decrease with the concentration of sugar [37,38,39,41], despite some studies reporting that no significant change in the enthalpy is observed [42]. Furthermore, this membrane-stabilizing effect is understood inductively as a common general property of sugars including monosaccharides, rather than a specific property based on the chemical structure of individual sugar molecules [38].

In this study, we examined the effect of four kinds of monosaccharides, namely, D-glucose, D-fructose, D-allose and D-psicose, on the bilayer phase transitions of DMPC by means of DSC. DSC is an experimental technique that has often been used in numerous previous studies on the phase transition behavior and thermodynamic properties of various phospholipid bilayer membranes and is also suitable for investigating the interactions of small molecules with phospholipid bilayer membranes on the basis of the above classification. Of all these four monosaccharides, D-allose [43] and D-psicose [44] are so-called rare sugars and correspond to the C-3 epimers of D-glucose and D-fructose, respectively. Since the bioproduction strategy for all rare sugars, commonly known as Izumoring [45], was established, the practical use of rare sugars has been developed, especially in the food and pharmaceutical industries. For this purpose, a wide range of biological studies have been performed so far to examine the physiological activity of rare sugars and their behavior in the human body. For example, it has been reported that absorbed D-psicose is not metabolized in the human body and more than 99% of it is excreted in the urine [46]. In addition, it has been revealed that D-psicose has the effect of lowering blood glucose levels through several mechanisms including the suppression of the absorption of D-glucose and D-fructose [46], and thus, D-psicose is strongly anticipated to be utilized as a zero-calorie sweetener to prevent and improve metabolic syndrome, such as in diabetes and obesity. As for D-allose, it has been reported that orally ingested D-allose is not metabolized and more than 90% of it is excreted in the urine within 24 h [47]. D-allose is also known to function as an antioxidant [48] and to have the effect of suppressing an increase in blood pressure [49] and of inhibiting the proliferation of cancer cells [50]. Therefore, D-allose is expected to be developed as a drug or quasi-drug rather than as a low-calorie sweetener. On the other hand, there have been very few studies on the physicochemical properties of these rare sugars relating to their interaction with cell membranes (e.g., molecular interaction with membrane lipids and membrane permeability). However, considering that there are many opportunities for these rare sugars to contact with various cell membranes (e.g., brush border membranes of intestinal epithelial cells) during the absorption process, we also need to have sufficient scientific knowledge about their physical and chemical behavior in the human body in order to make sure that their physicochemical properties induce no unexpected undesirable side-effects in the human body. It has recently been reported that sucralose, generally known as an artificial sweetener, induces the bilayer interdigitation [51], which indicates that slight modifications of the chemical structure of sugars can have a significant effect on the bilayer structure. As far as we know, this is the first report on the interaction between rare sugars and phospholipid bilayer membranes and will contribute to the further expansion of the practical utilization of rare sugars.

## 2. Materials and Methods

**Figure 1:**
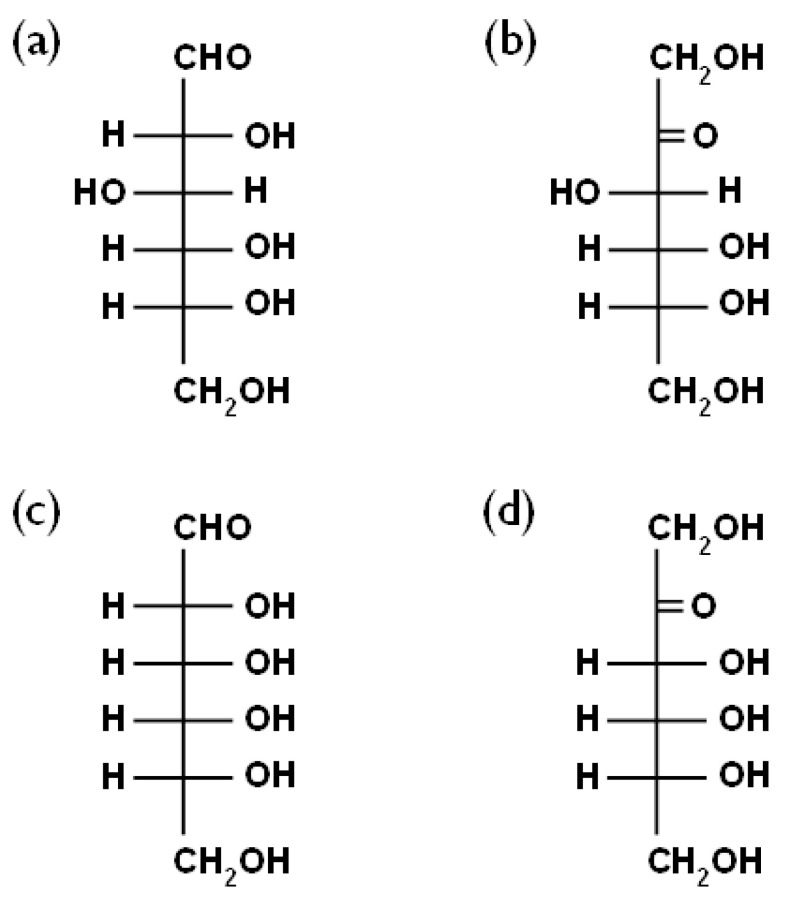
Chemical structures of (**a**) D-glucose, (**b**) D-fructose, (**c**) D-allose and (**d**) D-psicose.

A synthetic phosphatidylcholine, DMPC (1,2-dimyristoyl-*sn*-glycero-3-phospho- choline), was purchased from Avanti Polar Lipids (Alabaster, AL, USA). D-glucose and D-fructose were purchased from Kanto Chemical Co., Ltd. (Tokyo, Japan) and the rare sugars, D-allose and D-psicose, were provided from the International Institute of Rare Sugar Research and Education (Kagawa University). All the materials were used without further purification. The chemical structures of these monosaccharides are shown in Figure 1.

As the first step, an aqueous solution of each monosaccharide with a concentration of 3.30 mol kg^−1^ was prepared using Milli-Q water. This aqueous sugar solution was diluted with Milli-Q water to 1/5, 2/5, 3/5 and 4/5 to obtain a series of aqueous sugar solutions with different concentrations (i.e., 0.66, 1.32, 1.98 and 2.64 mol kg^−1^). An appropriate amount of each aqueous sugar solution was added to a weighed amount of DMPC powder, and the mixture was sonicated with a sonifier (Branson M3800-J, Emerson Electric Co., St. Louis, MO, USA) for a few minutes at 30 ± 1 °C, which is higher by at least 5 °C than the main-transition temperature of the DMPC bilayer membrane. Homogeneously translucent 1.0 mmol kg^−1^ DMPC vesicle suspensions in aqueous solutions of each sugar with different concentrations were finally obtained. In addition, 1.0 mmol kg^−1^ DMPC vesicle suspensions with an asymmetry in the sugar concentrations between the inside and the outside of the DMPC vesicle particles were prepared by the following procedure: First, a weighed amount of DMPC powder was dispersed into an appropriate amount of Milli-Q water by sonication to obtain 2.0 mmol kg^−1^ DMPC vesicle suspensions in water, and subsequently, the same amount of 2.64 mol kg^−1^ aqueous sugar solution (as Milli-Q water) was added to the 2.0 mmol kg^−1^ DMPC vesicle suspensions in water. Since all the sample dispersions were prepared through no process for controlling the particle size in this study, it is highly expected that they would contain vesicle particles with relatively large radii and that the distribution of the particle size would be wide.

Differential scanning calorimetry (DSC) measurements were carried out using a high-sensitivity differential scanning calorimeter VP-DSC (Malvern Instruments Ltd., Worcestershire, UK) at a heating rate of 0.75 °C min^−1^. DSC thermograms were recorded within the temperature range of 5–50 °C. A transition temperature was determined as the temperature at the top of an endothermic peak and an enthalpy change for the transition (Δ*H*) was estimated using Excel 2013 to integrate the endothermic peaks after the baseline subtraction to remove the contribution from the dispersion medium. The software ORIGIN ver. 7.0 that comes with the instrument was also used for comparability and confirmation of the validity of the baseline subtraction. We used several polynomial functions of different orders (from 3rd to 6th order) as fitting functions in Excel to obtain a calculated baseline to be used for baseline subtraction and confirmed that the choice of those functions would have no significant effect on the Δ*H* values finally obtained after the baseline subtraction.

## 3. Results and Discussion

### 3.1. Choice of Reference Solution and Stability of Baseline

In the first place, we confirmed the choice of a proper reference solution; that is, which of pure water or an aqueous solution of a corresponding monosaccharide is better to use as a reference solution to obtain more desirable results from the DSC measurements in this study. In principle, this choice of the appropriate reference solution has almost no effect on the accuracy and sensitivity of the DSC measurement itself, but the position of the baseline is affected by the difference in the heat capacity between the sample and the reference solution. Therefore, a proper choice of the reference solution is important for obtaining correct results in a DSC measurement. Here, pure water is considered preferable as a reference solution in the case where almost all the sugar molecules are incorporated into the DMPC bilayer membranes, whereas the same aqueous sugar solution is considered preferable when almost no sugar molecules are incorporated into the membranes.

**Figure 2:**
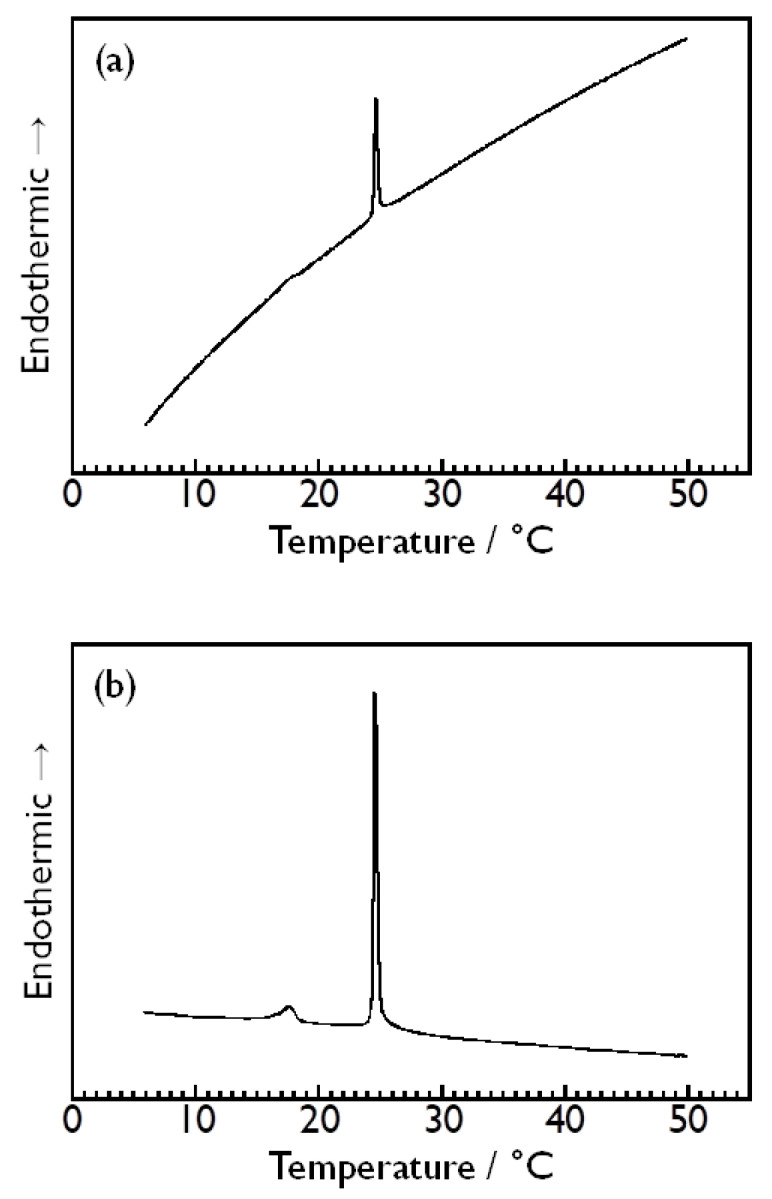
DSC thermogram obtained for DMPC bilayer membrane in aqueous solution of D-glucose with the concentration of 2.64 mol kg^−1^ by using (**a**) Milli-Q water or (**b**) the same aqueous D-glucose solution as a reference solution.

As an example, Figure 2a,b show the DSC thermograms obtained for the DMPC vesicle dispersion in the 2.64 mol kg^−1^ aqueous D-glucose solution in the case of using Milli-Q water as a reference solution or using the same 2.64 mol kg^−1^ aqueous D-glucose solution as was used for preparing the sample dispersion as a reference solution, respectively. Both thermograms exhibit a sharp endothermic peak at ca. 24 °C arising from the main transition of the DMPC bilayer membrane [1,2], which indicates that the DSC measurements performed in this study can detect this bilayer phase transition clearly, irrespective of the choice of which to use as a reference solution. However, it is evident that the stability of the baseline is completely different between both DSC thermograms. In the case of using Milli-Q water as a reference solution (Figure 2a), the stability of the baseline of the obtained thermogram is obviously poor (or low), and the whole range of the variation of the baseline throughout the measurement is apparently larger than the range of the variation at the endothermic peak (i.e., peak height) attributed to the main transition at 24 °C. This large variation in the baseline makes a small endothermic peak arising from the pretransition of the DMPC bilayer membrane at ca. 15 °C [1,2] almost indiscernible. Even if the presence of this small endothermic peak is discernible, it is quite difficult to determine the onset and the completion temperature of the peak accurately because of the large curvature of the baseline. This means that the DSC measurements performed on this condition is virtually unable to capture the pretransition of the DMPC bilayer membrane accurately. By contrast, the stability of the baseline is apparently far better in the DSC thermogram obtained when the aqueous D-glucose solution was used as a reference solution, and the variation in the baseline throughout the measurement is sufficiently small, as seen from Figure 2b. Note that this DSC thermogram is also a raw thermogram, as with that shown in Figure 2a, which means that both are compared under the same condition except for the difference in the reference solution used. This high stability of the baseline makes it easier to clearly identify the small endothermic peak arising from the pretransition, which enables us to determine the enthalpy change with the pretransition more correctly as well as the enthalpy change with the main transition. On the basis of this result, we used the same aqueous sugar solution as was used for preparing a sample solution as a reference solution in the subsequent DSC measurements to observe the phase transitions of the DMPC bilayer membrane in various kinds of aqueous sugar solutions. This fact, that the same aqueous sugar solution works much better as a reference solution, may suggest that in the sample dispersion (i.e., DMPC vesicles in the sugar solution), most of the monosaccharide molecules exist in the bulk water phase.

### 3.2. Bilayer Phase Transitions of DMPC in Aqueous Sugar Solutions

**Figure 3:**
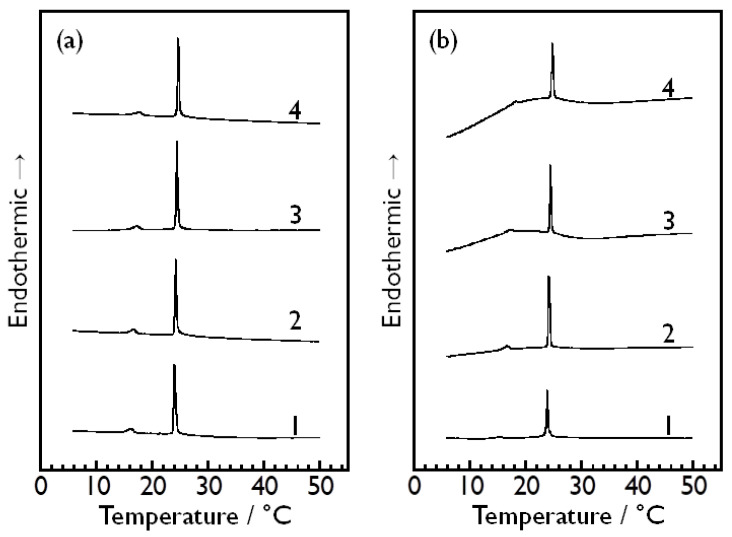

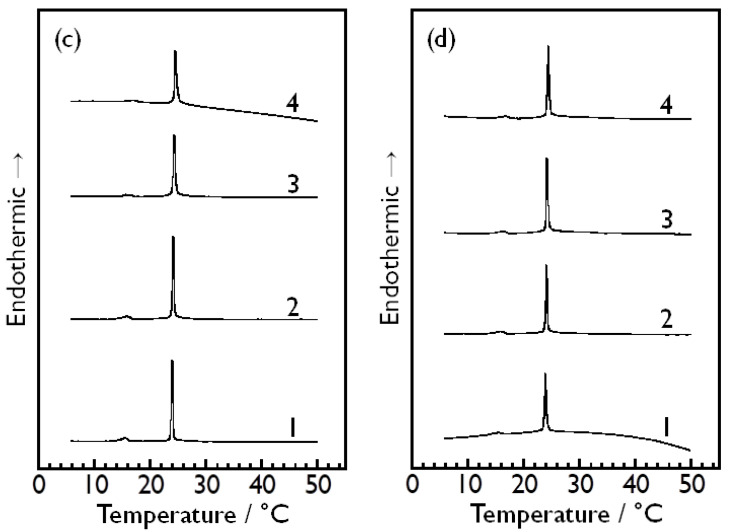
DSC thermograms obtained for DMPC vesicle dispersions in aqueous solutions of (**a**) D-glucose, (**b**) D-fructose, (**c**) D-allose and (**d**) D-psicose with different sugar concentrations (1: 0.66 mol kg^−1^, 2: 1.32 mol kg^−1^, 3: 1.98 mol kg^−1^, 4: 2.64 mol kg^−1^).

Figure 3 shows the DSC thermograms obtained for the DMPC vesicle dispersions in aqueous solutions of D-glucose (Figure 3a), D-fructose (Figure 3b), D-allose (Figure 3c) and D-psicose (Figure 3d) with different sugar concentrations (i.e., 0.66, 1.32, 1.98 and 2.64 mol kg^−1^ for each sugar). As described in the previous section, these DSC thermograms were obtained on the condition that the same aqueous sugar solution as used for preparing the sample solution was used as a reference solution, and thus, the stability of the baseline is sufficiently high in each thermogram with a few exceptions. As a result, the main transition of the DMPC bilayer membrane was successfully observed as a sufficiently sharp and large endothermic peak at ca. 24 °C in each thermogram, and the pretransition was also observed clearly as a small endothermic peak at ca. 15 °C in all the thermograms except those few exceptions, namely, the DSC thermograms for the DMPC bilayer membrane in the aqueous D-fructose solution of 1.98 mol kg^−1^ or 2.64 mol kg^−1^. We have confirmed that D-fructose-containing sample dispersions with relatively higher concentrations tend to exhibit an unstable baseline in the temperature range below about 20 °C, and that the manner of the variation in the baseline is not always the same. Currently, the reason for this has not been elucidated yet, but this may suggest that the partitioning of D-fructose to the DMPC bilayer membrane is easy to fluctuate as compared to those of the other monosaccharides or that there is a certain interaction between D-fructose molecules in the bulk water phase, which has an unstable effect on the heat capacity of the entire dispersion medium.

On the basis of the DSC results shown in Figure 3, we determined the pre- and main-transition temperatures, *T*_p_ and *T*_m_, of the DMPC bilayer membrane in each aqueous sugar solution with each concentration, and estimated the enthalpy change with the pre- and main transition, Δ*H*_p_ and Δ*H*_m_, by calculating the areas of the corresponding endothermic peaks in each DSC thermogram. Here, we should briefly mention the peak width at the half height, Δ*T*_1/2_, which is a physical quantity inversely related to the cooperativity of a transition in general. As is evident from Figure 3, the Δ*T*_1/2_ value did not significantly change irrespective not only of the sort of the sugars but also of their concentrations, which means that these sugars have virtually no effect on the cooperativity of the main transition of the DMPC bilayer membrane. This is consistent with a previous study by Crowe and Crowe [40], who reported that the presence of 1 M trehalose or glucose has virtually no effect on the size of the cooperative unit upon the main transition of the DPPC bilayer membrane in the case of multilamellar vesicles, though the cooperative unit significantly decreases with increasing concentration of trehalose, sucrose or fructose in the case with the DPPC extruded vesicles (i.e., unilamellar vesicles). In this study, therefore, we have not dealt with this kind of data as a significant index and no further discussion based on the Δ*T*_1/2_ values is given hereinafter.

**Figure 4:**
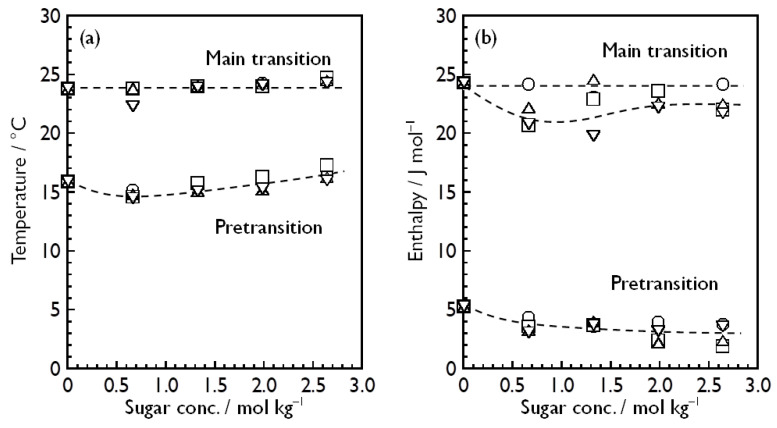
Sugar concentration dependence of (**a**) transition temperatures and (**b**) transition enthalpies of the pre- and main transition of DMPC bilayer membrane in the aqueous solution of each monosaccharide: D-glucose (circle), D-fructose (square), D-allose (triangle) and D-psicose (inverse triangle).

Figure 4 shows the sugar concentration dependence of the transition temperatures (Figure 4a) and transition enthalpies (Figure 4b) for each monosaccharide. As for the Δ*H* values, the same kind of plots with error bars (± standard deviation) for individual sugar-containing dispersions are given in the Supplementary Material (Figure S1). Strictly speaking, the *T*_m_ value elevated very slightly with increasing sugar concentration, as seen from Figure 4a, irrespective of the sort of monosaccharide. However, the degree of temperature elevation with increasing sugar concentration is small enough to regard *T*_m_ as almost constant, independent of the change in the sugar concentration. Some degree of variation in the transition temperature with increasing sugar concentration was also observed for the pretransition, but it is sufficiently small and, similarly, we may also regard *T*_p_ as almost constant, independent of the sugar concentration. Supposing that these small variations in *T*_p_ and *T*_m_ have some significance, this sugar concentration dependence of *T*_p_ and *T*_m_ may suggest that these monosaccharides tend to be partitioned most preferentially to the DMPC bilayer membrane in the L_β_′ phase, second preferentially to that in the P_β_′ phase and least preferentially to that in the L_α_ phase. This is a simple interpretation based on a thermodynamic equation which relates the change in the bilayer transition temperature Δ*T* to the difference in the partition coefficients between the respective phases Δ*K* [52]:

(1) $$\Delta T=\frac{RT^{2}\Delta K}{55.5\Delta H}$$

Here, *R* is the gas constant, and *T* and Δ*H* represent the pre- or main-transition temperature and the enthalpy change with the pre- or main transition for the pure DMPC bilayer membrane, respectively. Alternatively, another interpretation is possible to explain this increase in the bilayer transition temperatures from the standpoint of the HFE concept [32]. Assuming that sugar molecules tend to be excluded from the interfacial region of the bilayer membrane, the local osmotic imbalance arising from the exclusion of the sugar molecules will move hydrated water molecules away from the interfacial region, and as a result, a more condensed bilayer state (i.e., more compact conformation of the constituent lipids) is induced to minimize the area exposed to the solvent as much as possible. Although we cannot conclude here which interpretation is more likely to be true, it is interesting that these four kinds of monosaccharides have almost the same effect on the pre- and the main-transition temperature of the DMPC bilayer membrane. It may be reasonable to consider, at least, that the effect on the phase transitions of the DMPC bilayer membrane will be produced through some thermodynamic mechanisms like the general colligative properties of solutions rather than through specific direct interactions between DMPC and the respective monosaccharide molecules.

By contrast, it seems that the variations in the Δ*H*_p_ and Δ*H*_m_ values with increasing sugar concentration are somewhat large as compared to those in *T*_p_ and *T*_m_. As seen from Figure 4b, there is a clear tendency for the Δ*H*_p_ value to decrease slightly with increasing sugar concentration. It is generally known that when a third chemical species is added, the enthalpy change with the pretransition decreases with increase in its concentration, and finally, the pretransition is abolished in most cases. However, the pretransition was clearly observed as a definite phase transition with the enthalpy change of at least 2 kJ mol^−1^ even at the substantially higher sugar concentration of 2.64 mol kg^−1^, and thus, these monosaccharides are considered to be incapable of inducing the abolition of the pretransition. Considering the fact that the effect of these monosaccharides on the pretransition enthalpy is relatively small, this decrease in Δ*H*_p_ is presumed to be probably relevant to some change in the hydration state around the polar headgroups caused by the presence of monosaccharide molecules. In addition, taking into account that all kinds of sugar-containing DMPC vesicle dispersions exhibit similar decreasing tendencies of Δ*H*_p_ regardless of the sort of the monosaccharide, it is unlikely that this decrease in Δ*H*_p_ is brought about by specific direct interaction between the DMPC and each kind of monosaccharide molecule. On the other hand, the sugar concentration dependence of Δ*H*_m_ seems more complicated. The Δ*H*_m_ value is almost constant independent of the sugar concentration for the D-glucose-containing system, whereas it decreases with increasing sugar concentration up to a certain concentration, but conversely increases above the concentration and finally is almost constant for the other sugar-containing systems. This means that there is a slight but definite difference in the sugar-concentration dependence of Δ*H*_m_ between the D-glucose-containing system and the other sugar-containing systems, and therefore, it may seem reasonable to consider that some specific direct interaction between the DMPC and monosaccharide molecules is involved in this variation in Δ*H*_m_ with increasing sugar concentration. However, this is still unlikely because although a relatively large amount of monosaccharide molecules exist in the system, the effect of them is too small (only a decrease of at most 5 kJ mol^−1^ in Δ*H*_m_) as compared to the cases of other additives, such as alcohols [18], fatty acids [17] and anesthetics [17], to assume that this is caused by specific direct interaction with the DMPC molecules. Considering all the data obtained from the DSC measurements, therefore, we may conclude that there is no direct interaction between the DMPC and each of these monosaccharide molecules. This is also consistent with the relation between the choice of the reference solution and the stability of the baseline which suggests that most of the monosaccharide molecules exist in the bulk water phase, because this inversely means that only a small amount of monosaccharide molecules can interact with the DMPC molecules.

### 3.3. Effect of Asymmetry in Sugar Concentration Between Inside and Outside of Vesicle Particles on Bilayer Phase Transitions of DMPC

**Figure 5:**
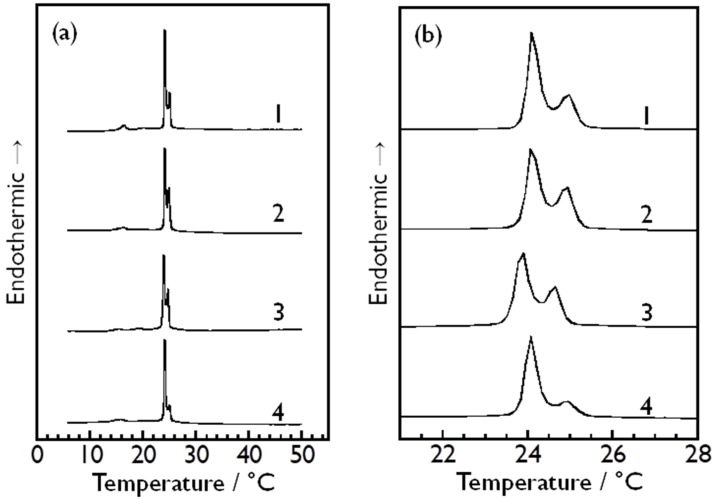
(**a**) Overall DSC thermograms obtained using unusual sugar-containing sample dispersions of DMPC vesicle particles with sugar concentration asymmetry between the inside and the outside of vesicle particles (inside: 0 mol kg^−1^; outside: 1.32 mol kg^−1^) for each kind of monosaccharide: 1: D-glucose, 2: D-fructose, 3: D-allose, 4: D-psicose. (**b**) Magnified view of the temperature region between 21 °C and 28 °C of each overall thermogram shown in the panel (**a**).

As described above, the effect of these monosaccharides on the bilayer phase transitions of DMPC was too small to obtain significant information about its molecular mechanism through conventional DSC measurements. Taking this into consideration, we prepared somewhat unusual sample dispersions containing each sugar, where the monosaccharide molecules are present only outside the DMPC vesicle particles (i.e., in the bulk water phase). In these samples, it is only the outer monolayer of the outermost bilayer membrane of each multilamellar DMPC vesicle that the monosaccharide molecules can interact with, because they are not able to pass through phospholipid bilayer membranes, in general. If the DMPC bilayer membranes affected by the presence of monosaccharide molecules and those being subject to no influence of monosaccharides undergo the phase transitions at different temperatures, it may be possible to detect both phase transitions simultaneously as two separate peaks in a single DSC scan by using this type of sugar-containing sample dispersion. Figure 5a shows the overall DSC thermograms obtained using this type of DMPC vesicle dispersion, containing each of D-glucose (curve 1), D-fructose (curve 2), D-allose (curve 3) and D-psicose (curve 4). Each thermogram shown in Figure 5b corresponds to a magnified view of the temperature range of 21–28 °C of each overall DSC thermogram shown in Figure 5a. As evident from Figure 5b, the endothermic peak arising from the main transition of the DMPC bilayer membrane split into two peaks, suggesting that the presence of each of these monosaccharides has a certain effect on the main transition of the DMPC bilayer membrane. However, considering that each sugar-containing dispersion exhibits similar peak-splitting behavior, and moreover, that the temperature difference between the two peaks for each sugar-containing dispersion is close to one another (i.e., 0.76–0.89 °C), it is not reasonable to interpret this peak-splitting behavior as indicating the presence of a specific interaction with the DMPC molecules, depending on its molecular structure. Currently, we assume two possibilities as to what causes the split of the endothermic peak as below.

The first possibility is that the split of the endothermic peak is due to the asymmetry of the interaction of the monosaccharide molecules with the DMPC molecules in the inner and the outer monolayer of the outermost DMPC bilayer membrane. This is exactly what we expected for this unusual sample dispersion with the concentration asymmetry between the inside and outside of the DMPC vesicle particles. That is, since there are no monosaccharide molecules inside the DMPC vesicle particles, no interaction between the DMPC and monosaccharide molecules can occur in the inner monolayer, but it can in the outer monolayer. If this asymmetry of the molecular interaction between the inside and the outside of the outermost bilayer membrane gives rise to a slight difference in the main-transition temperature between the inner and outer monolayer, it is possible that the DMPC bilayer membrane undergoes the main transition twice at two different temperatures. Note that the interaction we assume here is not specific direct molecular interaction, as described above, but an indirect one that can produce an effect on the bilayer phase transition like the thermodynamic colligative properties of solutions. For example, suppose that this split of the endothermic peak is due to the elevation of the main-transition temperature brought about by the boiling point elevation. Here, we should note that the presence of each monosaccharide induces increases in *T*_m_. Following this assumption, a simple calculation with the general thermodynamic equation for the boiling point elevation (Equation (2)) yields the following numerical result: the mole fraction of the monosaccharide molecules interacting with the DMPC molecules is 0.021–0.025.

(2) $$\ln\left(1-X_{S}\right)=\frac{\Delta H_{m}{}^{\circ}}{R}\left(\frac{1}{T_{m}}-\frac{1}{T_{m}{}^{\circ}}\right)$$

Here, *T*_m_° and Δ*H*_m_° represent the main-transition temperature and the enthalpy change with the main transition for the pure DMPC bilayer membrane, respectively, and *T*_m_ and *X*_S_ represent the main-transition temperature of the DMPC bilayer membrane containing monosaccharide molecules and the mole fraction of the monosaccharide within the bilayer membrane. Since the preparation concentration of DMPC is 1.0 mmol kg^−1^, this result means that the concentration of the monosaccharide interacting with the DMPC molecules is at most 25 μmol kg^−1^, which is equivalent to no more than 0.0019% of the total monosaccharide molecules present in the system. Apparently, this result seems to support the above described speculation that most of the monosaccharide molecules exist in the bulk water phase, but we should be cautious about the interpretation of this value, because this value is more likely to indicate the difference between the partition coefficients of these monosaccharides to the gel-state bilayer membrane and the liquid-crystalline-state bilayer in the real system, rather than the absolute value of the mole fraction of the monosaccharides present in the bilayer membrane. Actually, this *X*_S_ value is evidently smaller than similar kinds of values reported in previous studies (e.g., *K*~0.5 for glucose [32] and ca. 0.07 from the Γ_3_ value for sucrose and trehalose [36]).

As the second possibility, we assume that the split of the endothermic peaks is caused by changes in the DMPC bilayer structure due to the osmotic pressure generated by the difference in the sugar concentration between the inside and the outside of the vesicle particles. In this case, since the osmotic pressure gives rise to the transfer of water molecules from the inside to the outside of the DMPC vesicle particle, the particles can shrink inward. If the shape of the DMPC vesicle particles is largely distorted by the shrinkage, many regions with different curvatures will occur within a bilayer membrane. Since the molecular packing of a bilayer membrane tends to be looser in a region with a relatively large curvature and vice versa in general, the presence of various regions with largely different curvatures might be responsible for the split of the endothermic peak because the main transition is expected to occur at a higher temperature in a region with tighter molecular packing and at a lower temperature in a region with looser molecular packing. Even if the osmotic pressure does not actually produce an appreciable change in the shape of the DMPC vesicle particles, it can still cause the split of the endothermic peak arising from the main transition. In this case, since the shape of the DMPC vesicle particle does not change, the situation where there is a pressure difference due to the osmotic pressure between the inside and the outside of each vesicle particle is very similar to the situation where hydrostatic pressure of the same magnitude is externally applied to each vesicle particle. Note that this similarity in both situations is only applicable to the outermost bilayer membrane of a multilamellar vesicle, because the osmotic pressure generates the pressure difference only between the inside and the outside of the outermost bilayer membrane. Assuming that this similarity holds true, we can easily estimate the main-transition temperature of the outermost DMPC bilayer membrane. In our previous study [2], the pressure dependence of the main-transition temperature was determined to be 0.212 K MPa^−1^ for the DMPC bilayer membrane. In addition, the magnitude of the pressure difference due to the osmotic pressure Π in this sugar-containing sample dispersion is calculated to be 3.26 MPa using the general thermodynamic equation with respect to the osmosis (i.e., the van’t Hoff equation):

(3) $$\Pi =c_{S}RT$$

Here, *c*_S_ represents the molarity of a monosaccharide in the bulk water phase. With these data, we can presume the main-transition temperature of the outermost DMPC bilayer membrane in this sugar-containing sample to be higher by 0.69 °C than that of the other inner bilayer membranes that are assumed to exist without being affected by the osmotic pressure. It is interesting that although this calculation result of 0.69 °C was obtained through the rough calculation based on the rough assumption, it is close to the temperature difference between the two endothermic peaks (0.76–0.89 °C) that were observed in the DSC thermograms for the sugar-containing DMPC vesicle dispersions with the concentration asymmetry.

Finally, we briefly summarize the thermodynamic consideration of the peak-splitting behavior observed for the unusual sugar-containing sample dispersions with a concentration asymmetry between the inside and the outside of vesicle particles. As described above, we proposed two possibilities based on the commonly known colligative properties of solutions, namely, freezing point depression and osmotic pressure, to explain this peak-splitting behavior. Qualitatively, both thermodynamic effects can explain the observed peak-splitting behavior. However, the latter effect is expected to be more likely to have caused this peak-splitting behavior from a quantitative point of view, because our rough calculation for the degree of the transition-temperature elevation by the osmotic pressure on the basis of the pressure effect on the transition temperature was in relatively good agreement with the temperature difference between the split peaks. Here, we should note that very rough assumptions are made to calculate the degree of the transition temperature elevation and also that those assumptions are based on an extremely simplified (or idealized) situation. Further detailed investigations by means of other experimental techniques, especially spectroscopic techniques such as NMR [53,54] and FT-IR [55,56], are required to prove that this peak-splitting behavior is truly attributed to osmotic pressure.

## 4. Conclusions

In this study, we observed the bilayer phase transitions of DMPC in in aqueous solutions of four kinds of monosaccharides, namely, D-glucose, D-fructose, D-allose and D-psicose, by means of DSC measurements to investigate the effect of these monosaccharides with different molecular structures on the bilayer phase transitions of DMPC. We first confirmed the choice of a proper reference solution, that is, which of pure water or an aqueous solution of a corresponding monosaccharide is better to use as a reference solution for obtaining better DSC results. By comparing the DSC thermograms obtained in both cases, we found that the same aqueous sugar solution as used for preparing a sugar-containing sample dispersion should be used as a reference solution to obtain better DSC results with high stability of the baseline. This suggests the possibility that most of the monosaccharide molecules exist in the bulk water phase. We next performed the DSC measurements using two different types of sugar-containing sample dispersions to investigate the effect of the monosaccharides on the bilayer phase transitions in detail. For each kind of monosaccharide, one is a normal type of sugar-containing sample dispersion with no difference in the sugar concentrations of the inside and the outside of the DMPC vesicle particles, while the other is an unusual type of sugar-containing sample dispersion with a concentration asymmetry between the inside and the outside of the vesicle particles. From a series of DSC measurements using the normal sugar-containing sample dispersions with different sugar concentrations, it was found that the thermodynamic properties of the pre- and main transitions of the DMPC bilayer membrane (i.e., *T*_p_, *T*_m_, Δ*H*_p_ and Δ*H*_m_) hardly varied with increasing sugar concentration, and moreover, that there is no significant difference in the concentration dependence of these properties among these four kinds of monosaccharides. This suggests that only a very small number of monosaccharide molecules can interact with the DMPC bilayer membrane, and moreover, that the interaction between the DMPC and each kind of monosaccharide molecules is not a specific one depending on the molecular structure of each kind of monosaccharide.

The DSC thermograms obtained using the unusual sugar-containing sample dispersions demonstrated that the concentration asymmetry caused the endothermic peak arising from the main transition of the DMPC bilayer membrane to split into two endothermic peaks. This peak-splitting behavior was commonly observed for all the sample dispersions containing each monosaccharide, and the temperature difference between the two peaks was almost the same (0.76–0.89 °C) irrespective of the sort of monosaccharide present in the dispersion. This common feature indicates the possibility that this peak-splitting behavior was brought about through a thermodynamic mechanism, like the general colligative properties of solutions, rather than by specific direct interaction between DMPC and respective monosaccharide molecules. We assumed two possible thermodynamic reasons for this split of the endothermic peak: one is freezing point depression and the other is osmotic pressure. Qualitatively, both can explain the peak-splitting behavior, but our numerical considerations based on the latter reason provided a quantitative agreement about the temperature difference between the split two peaks. Therefore, it may be reasonable to presume that the splitting of the endothermic peak occurred as a result of the elevation of the main-transition temperature of only the outermost DMPC bilayer membrane due to the osmotic pressure generated by the concentration asymmetry. Further detailed investigation is necessary for identifying what causes the splitting of the endothermic peak of the main transition, and we will continue to study the effect of monosaccharides, especially rare sugars, on the bilayer phase transitions to obtain the chemical and physical knowledge for better understanding the biological features and behavior of rare sugars in our body. Such scientific knowledge will surely help not only to promote the wide use of rare sugars as food and medicines but also to develop novel ways to utilize rare sugars.

## Data Availability

The raw data supporting the conclusions of this article will be made available on request from the corresponding author due to privacy.

## Supplementary Materials

The following supporting information can be downloaded at: https://www.mdpi.com/article/10.3390/membranes14120258/s1, Figure S1: Sugar concentration dependence of transition enthalpies of the pre- and main transition of DMPC bilayer membrane in the aqueous solution of (a) D-glucose, (b) D-fructose, (c) D-allose and (d) D-psicose.
