# Supplementary material for: Effect of Monosaccharides Including Rare Sugars on the Bilayer Phase Behavior of Dimyristoylphosphatidylcholine
Source: Membranes (Basel). 2024 Dec 3;14(12):258. doi: 10.3390/membranes14120258 (PMC11676506; doi:10.3390/membranes14120258)
Supplement: Supplementary file 1 [file membranes-14-00258-s001.zip › membranes-3212198-supplementary.pdf]

Supplmentary Materials for:

Effect of Monosaccharides Including Rare Sugars on the Bilayer Phase Behavior of Dimyristoylphosphatidylcholine

Nobutake Tamai[1], Mei Kamiya[2], Nono Kiriyama[2], Masaki Goto[1],
Kazuhiro Fukada[3] and Hitoshi Matsuki[1],*

1 Department of Bioengineering, Division of Bioscience and Bioindustry, Graduate School of Technology, Industrial and Social Sciences, Tokushima University, 2-1 Minamijosanjima-cho, Tokushima 770-8513, Japan.
2 Department of Applied Life Science, Division of Bioresource Science, Graduate School of Sciences and Technology for Innovation, Tokushima University, 2-1 Minamijosanjima-cho, Tokushima 770-8513, Japan.
3 Department of Applied Biological Science, Faculty of Agriculture, Kagawa University, 2393 Ikenobe, Miki-cho, Kita district, 761-0795, Japan.

*To whom correspondence should be addressed:
E-mail: matsuki@tokushima-u.ac.jp
Phone: +81 88 656 7513
Fax : +81 88 655 3162

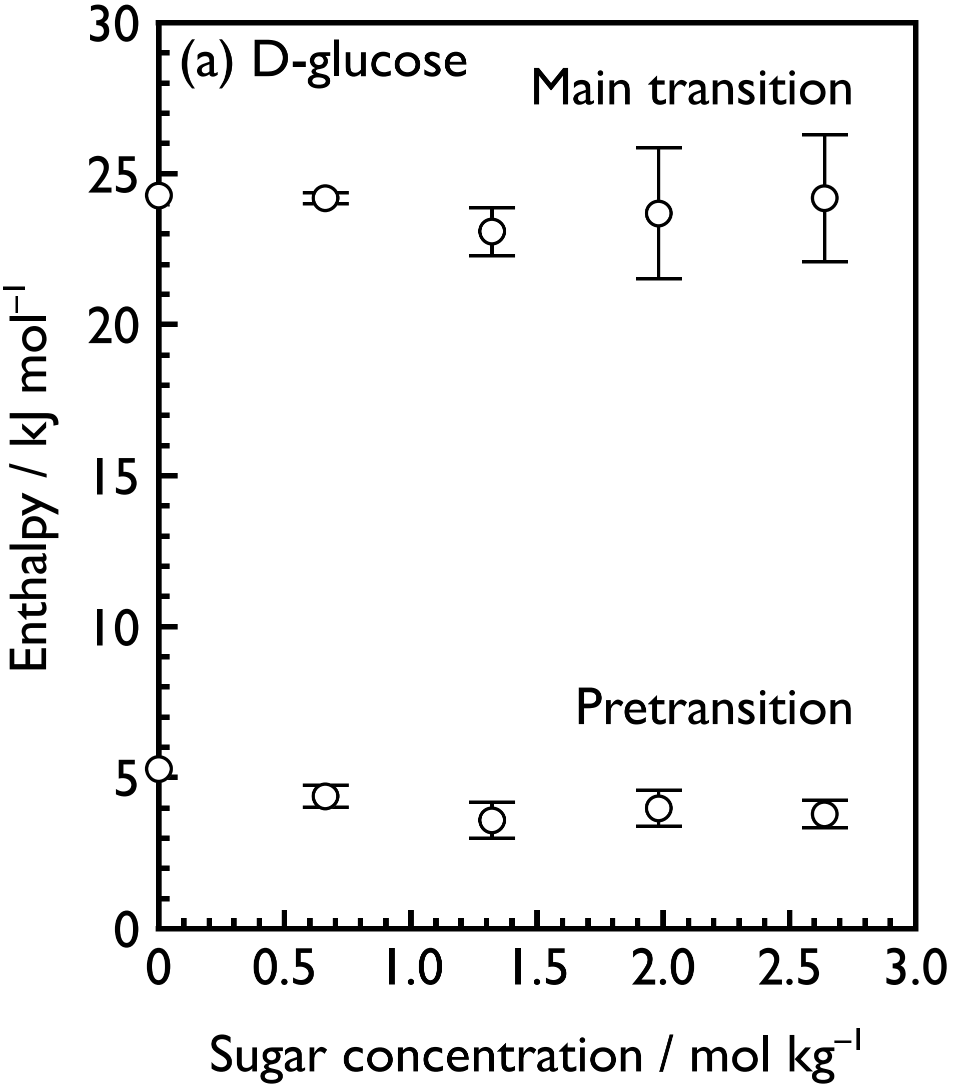

(a) D-glucose
Main transition
Pretransition
Enthalpy / kJ mol⁻¹
Sugar concentration / mol kg⁻¹
30
25
20
15
10
5
0
0
0.5
1.0
1.5
2.0
2.5
3.0

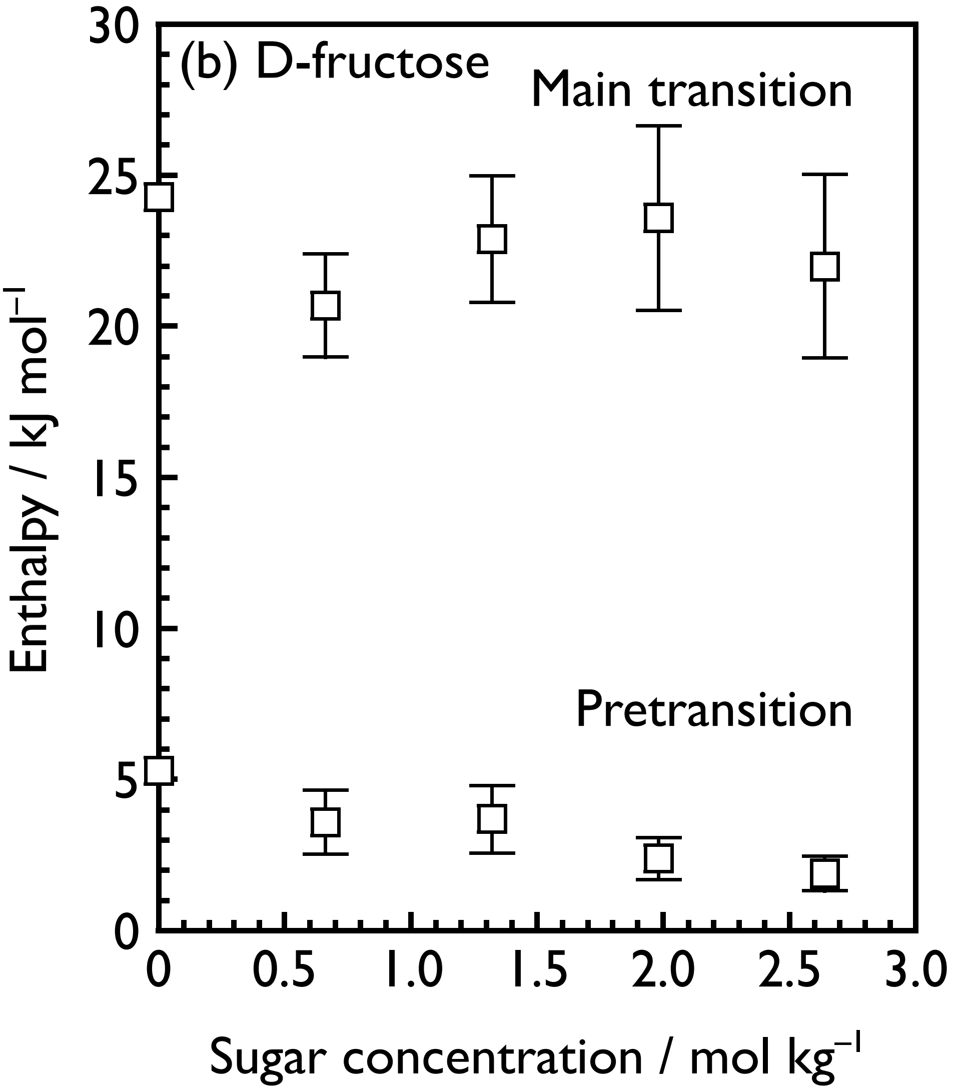
(b) D-fructose
Main transition
Pretransition
Enthalpy / kJ mol^{-1}
Sugar concentration / mol kg^{-1}
0
5
10
15
20
25
30
0
0.5
1.0
1.5
2.0
2.5
3.0

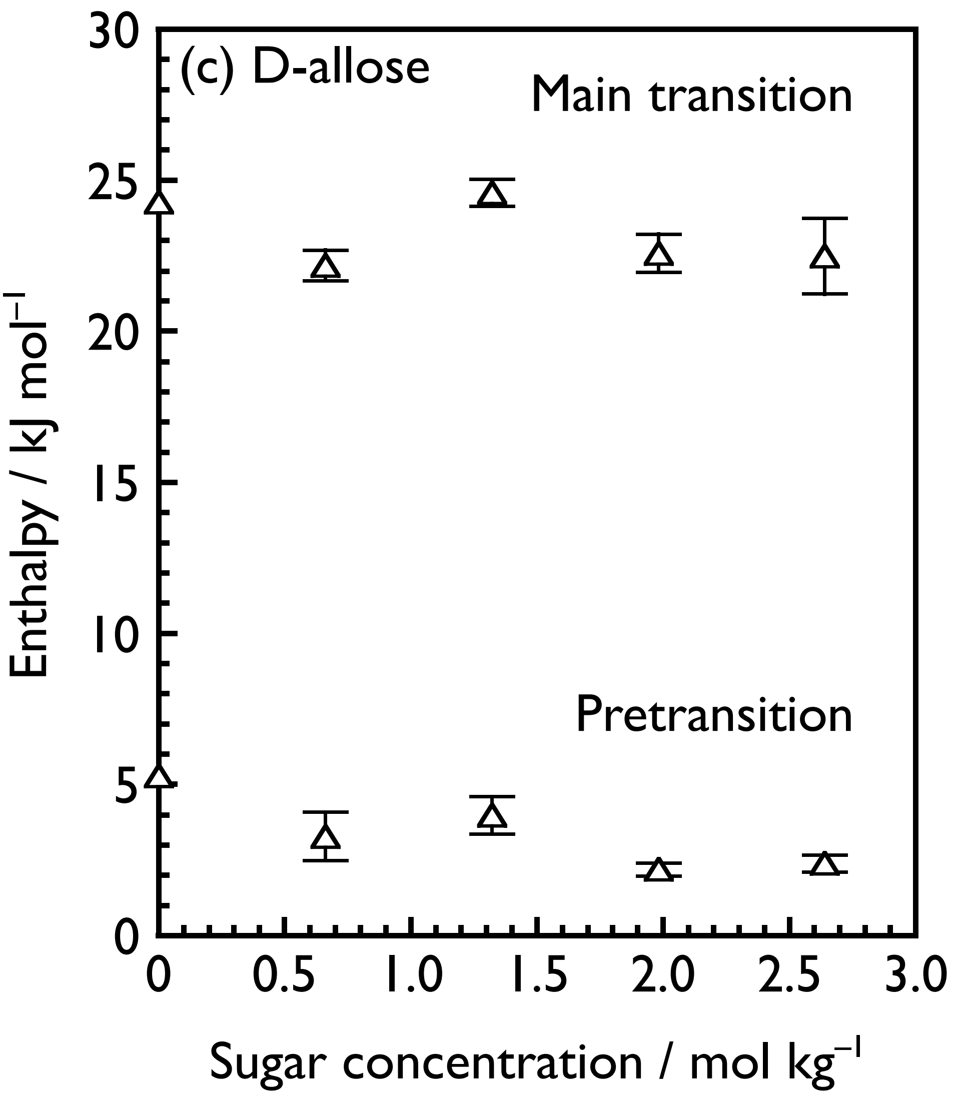

(c) D-allose
Main transition
Pretransition
Enthalpy / kJ mol⁻¹
Sugar concentration / mol kg⁻¹
0
5
10
15
20
25
30
0
0.5
1.0
1.5
2.0
2.5
3.0

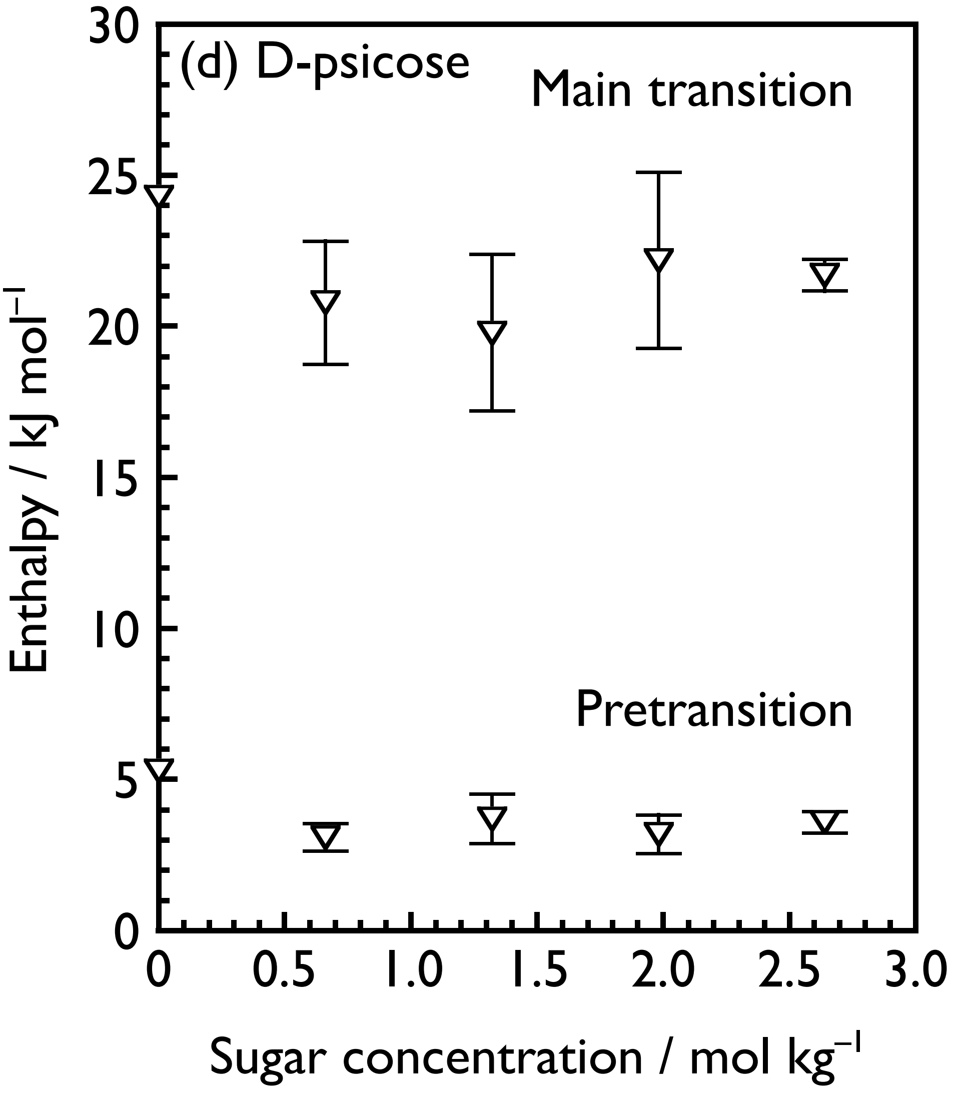

Figure S1. Sugar concentration dependence of transition enthalpies of the pre- and main transition of DMPC bilayer membrane in the aqueous solution of (a) D-glucose, (b) D-fructose, (c) D-allose and (d) D-psicose. Each pair of error bars on each plot represents the standard deviation. Except for the presence or absence of those error bars, the data shown here are complete identical to those given in Figure 4 (b) in the text.
